# Circulating IncRNA DRAIC as a potential serum biomarker for predicting lymphatic and vascular spread in pancreatic cancer

**DOI:** 10.5937/jomb0-63713

**Published:** 2026-05-22

**Authors:** Ying Che, Ting Zhang, Jing Li, Xiao Jiang, Chao Li, Wei Li

**Affiliations:** 1 Beijing Anzhen Hospital Affiliated to Capital Medical University, Department of Gastroenterology, Beijing, China; 2 Zhongshan Hospital, Fudan University, Department of Gastroenterology, Shanghai, China

**Keywords:** pancreatic cancer, DRAIC, vascular involvement, lymph node metastasis, receiver operating characteristic curve, karcinom pankreasa, DRAIC, vaskularna invazija, metastaze u limfnim čvorovima, ROC kriva

## Abstract

**Background:**

Pancreatic cancer is notorious for its aggressive behavior and poor prognosis, largely due to delayed diagnosis and early dissemination. Identifying reliable serum biomarkers could enhance early detection and risk stratification. Long non-coding RNAs (lncRNAs) have been recognised as regulators of tumour biology; however, the clinical and biochemical relevance of lncRNA DRAIC in pancreatic cancer remains unclear.

**Methods:**

Serum samples were obtained from 100 patients diagnosed with pathologically confirmed pancreatic cancer and 100 ageand sex-matched controls. Quantitative real-time polymerase chain reaction (qPCR) was employed to quantify the circulating DRAIC expression levels. We analysed the associations between serum DRAIC levels and clinicopathological characteristics, including vascular involvement and lymph node metastasis. Receiver operating characteristic (ROC) curve analyses were performed to assess the diagnostic and predictive performance of DRAIC. Paired serum samples obtained before and after surgical resection were additionally analysed.

## Abstract (sr)

**Uvod:**

Karcinom pankreasa je poznat po svom agresivnom toku i lošoj prognozi, uglavnom zbog kasne dijagnoze i ranog širenja bolesti. Identifikacija pouzdanih serumskih biomarkera mogla bi da unapredi rano otkrivanje i stratifikaciju rizika. Duga nekodirajuća RNK (lncRNA) prepoznata je kao regulator tumorske biologije, međutim klinički i biohemijski značaj lncRNA DRAIC kod karcinoma pankreasa još uvek nije u potpunosti razjašnjen.

**Metode:**

Prikupljeni su uzorci seruma od 100 pacijenata sa patohistološki potvrđenim karcinomom pankreasa i 100 kontrolnih pacijenata usklađenih po starosti i polu. Kvantitativna lančana reakcija polimeraze u realnom vremenu (qPCR) je korišćena za određivanje nivoa cirkulišuće ekspresije DRAIC. Analizirane su povezanosti između serumskih nivoa DRAIC i kliničko-patoloških karakteristika, uključujući vaskularnu invaziju i metastaze u limfnim čvorovima. Analize ROC kriva su sprovedene radi procene dijagnostičkih i prediktivnih performansi DRAIC. Dodatno su analizirani upareni uzorci seruma dobijeni pre i posle hirurške resekcije.

**Rezultati:**

Ekspresija DRAIC u serumu je bila značajno povišena kod pacijenata sa karcinomom pankreasa u poređenju sa kontrolnim pacijentima. ROC analiza je pokazala snažan dijagnostički potencijal (AUC=0,943). Povišeni nivoi DRAIC bili su značajno povezani sa metastazama u limfnim čvorovima i vaskularnom invazijom. DRAIC je pokazao visoku prediktivnu vrednost za metastaze u limfnim čvorovima (AUC=0,906), dok je njegova prediktivna vrednost za vaskularnu invaziju bila umerena. Značajno je i da su serumski nivoi DRAIC bili značajno sniženi nakon hirurške resekcije.

**Zaključak:**

Cirkulišuća lncRNA DRAIC je bitno deregulisana kod karcinoma pankreasa i pokazuje potencijalnu primenu kao serumski biohemijski biomarker, naročito u proceni metastaza u limfnim čvorovima. Ovi nalazi predstavljaju osnovu za dalja mehanistička istraživanja i validaciju na većim kohortama.

## Introduction

Pancreatic ductal adenocarcinoma (PC) ranks among the most aggressive cancers worldwide [1][2]. Despite advancements in surgical approaches and systemic therapies, the prognosis for PC remains dismal, with a reported 5-year survival rate near 7% and a median survival of less than nine months [3][4]. Early-stage diagnosis followed by appropriate surgical intervention and adjuvant chemotherapy is currently the most effective strategy for improving patient outcomes [5]. However, due to the absence of sensitive and reliable biochemical and molecular markers for early detection and metastatic risk assessment, most patients presented at advanced stages, resulting in limited therapeutic options and an unfavourable prognosis. Therefore, identifying novel molecular indicators with diagnostic and prognostic value is a critical challenge in pancreatic cancer research.

In recent years, non-coding RNAs have gained attention as key modulators of gene expression and cellular processes. Long non-coding RNAs (lncRNAs) participate in diverse biological mechanisms, including transcriptional modulation, post-transcriptional processing, epigenetic modification, and intracellular signalling [6][7][8]. Notably, aberrant lncRNA expression has been reported in malignancies of the digestive, respiratory, urinary, and reproductive systems, highlighting their potential value as biochemical and molecular biomarkers [9][10][11][12][13][14][15].

In pancreatic cancer, emerging evidence suggests that lncRNAs are involved in tumour progression and metastatic dissemination. Tahira et al. [16] identified significant differential expression of 134 lncRNAs in metastatic PC tissues, suggesting a potential role of lncRNAs in distant metastasis. These findings underscore the importance of exploring circulating and tissue-derived lncRNAs as candidate biomarkers for pancreatic cancer diagnosis and prognostic stratification.

DRAIC (LOC145837) is a 1.7 kb non-coding RNA located on chromosome 15q23, adjacent to another non-coding RNA gene, PCAT29 [17]. Previous research has shown aberrant DRAIC expression in various cancers, suggesting that it functions as an oncogenic regulator. In breast cancer, elevated DRAIC expression has been shown to promote tumour cell proliferation, and higher DRAIC mRNA levels have been detected in patient samples [18]. Nevertheless, the clinical and biochemical relevance of DRAIC in pancreatic cancer has not yet been investigated.

Based on these observations, the present study investigates the expression of circulating lncRNA DRAIC in the serum of PC patients and to analyse its association with clinicopathological features, particularly vascular involvement and lymph node metastasis. By assessing its diagnostic and predictive performance using receiver operating characteristic analysis, this study seeks to clarify the potential value of DRAIC for pancreatic cancer, thereby providing additional laboratory evidence to support its clinical application.

## Materials and methods

### Clinical information

A total of 100 patients diagnosed with pancreatic cancer (PC) at our hospital were enrolled in this study. The patient cohort included 44 men and 56 women, with an average age of 61.09±5.23 years. During the same period, 100 healthy individuals undergoing routine physical examination were recruited as the control cohort (46 men, 54 women; mean age 59.3±4.9 years). No significant difference in age or sex distribution was observed between the two groups (P>0.05), indicating baseline comparability.

### Serum sample collection and processing

Fasting venous blood samples (3 mL) were collected in the morning and allowed to clot at room temperature. Samples were centrifuged at 3,000 rpm for 10 minutes within a standardised processing window to separate serum. The supernatant serum was immediately aliquoted to avoid repeated freeze-thaw cycles and stored at -20°C for short-term preservation prior to RNA extraction. All samples from patients and controls underwent identical pre-analytical handling to minimise systematic variability in circulating RNA measurements.

### RNA extraction and quantitative real-time PCR

Total RNA was extracted from serum using TRIzol reagent (Invitrogen, Carlsbad, CA, USA) following the manufacturer's instructions. RNA quantity and purity were assessed before downstream processing. Complementary DNA (cDNA) was synthesised from the total RNA. qPCR was performed using 2x SYBR Green PCR Master Mix (Bao Biological Engineering, Dalian, China) under standard cycling conditions. GAPDH served as the internal reference for normalisation. Primer sequences were as follows: lncRNA DRAIC: Forward: 5'-TGAACTCAACTCCTGAGAAGGAC-3 '; Reverse: 5'-CGCTCTCAGACTCTTCAGTTCTC-3'; GAPDH: Forward: 5'-CTCTGCTCCTCCTGTTCGAC-3'; Reverse: 5'-TTAAAAGCAGCCCTGGTGAC-3'. Relative expression levels were determined using standard comparative quantification methods.

### Statistical analysis

All statistical computations were conducted with SPSS 20.0 (IBM, Armonk, NY, USA). Continuous data are shown as mean ± SD. Group comparisons were performed using t-tests for two-group analyses and one-way ANOVA for multiple-group comparisons. Receiver operating characteristic (ROC) curves were constructed to assess the diagnostic and predictive utility of serum DRAIC for pancreatic cancer, lymph node metastasis, and vascular involvement. Area under the curve (AUC), sensitivity, and specificity were measured. A P-value<0.05 was set as statistically significant.

## Results

### Serum DRAIC expressions

**Figure 1:**
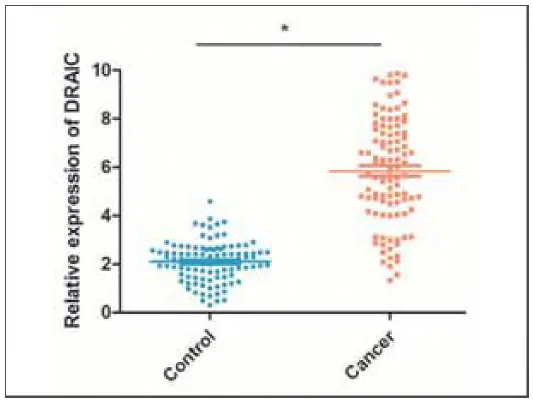
Comparison of DRAIC between pancreatic cancer patients and healthy controls. The expression of DRAIC in patients with pancreatic cancer detected by RT-PCR was significantly higher than that in healthy people, and the difference was statistically significant (P < 0.05).

Quantitative real-time PCR analysis demonstrated that serum DRAIC expression levels were statistically elevated in patients with pancreatic cancer compared with healthy people (P<0.05) (Figure 1). This result indicates a marked dysregulation of circulating DRAIC in pancreatic cancer.

### Diagnostic performance of serum DRAIC for pancreatic cancer

**Figure 2:**
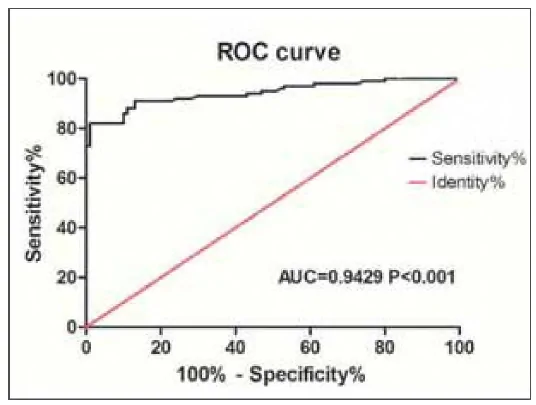
The predictive power of serum DRAIC for pancreatic cancer. When DRAIC predicted the occurrence of pancreatic cancer, the area under the ROC curve was 0.9429, P < 0.001, and the sensitivity was 91%, and the specificity was 85% when the cutoff value was 2.765.

The area under the ROC curve (AUC) was 0.943 (P<0.001). Using a cutoff value of 2.765, the sensitivity and specificity were 91% and 85%, respectively (Figure 2), indicating high diagnostic accuracy.

### Association between serum DRAIC levels and clinicopathological characteristics

We examined the association between serum DRAIC levels and clinical characteristics in 100 patients with pancreatic cancer. Elevated DRAIC expression was significantly linked to vascular involvement and lymph node metastasis (P<0.05). No significant associations were observed between DRAIC levels and patient sex, age, tumour location, or TNM stage (P>0.05) (Table 1).

**Table 1:** Clinicopathological characteristics in the enrolled patients with pancreatic cancer.

Variable	n	x̄±s	*t*	*P*
sex				
male	44	5.42±1.21	1.302	0.196
female	56	5.76±1.36		
age				
<60	57	4.78±1.46	1.346	0.181
≥60	43	5.21±1.73		
Tumour location				
Pancreatic head cancer	48	4.41±2.17	0.949	0.345
Pancreatic body and tail cancer	52	4.86±2.54		
TNM staging				
I + II	55	4.37±2.06	1.535	0.128
III + IV	45	4.98±1.87		
Vascular involvement				
Yes	39	6.83±2.76	2.178	0.032
No	61	5.78±2.05		
Lymph node metastasis				
Yes	49	6.98±3.21	5.72	<0.001
No	51	3.88±2.12		

### Predictive value of serum DRAIC for vascular involvement and lymph node metastasis

**Figure 3:**
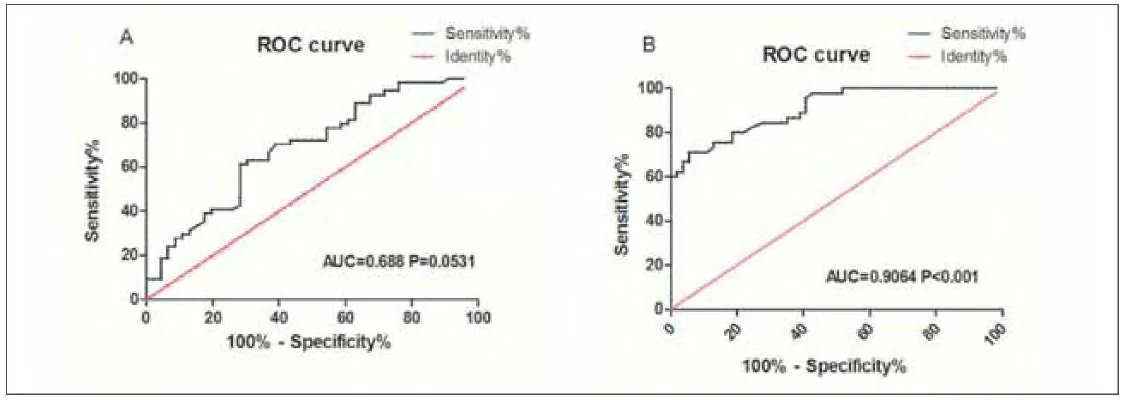
DRAIC evaluation of clinical diagnosis of vascular involvement and lymph node metastasis in patients with pancreatic cancer. (A) The area under the ROC curve for serum DRAIC level in evaluating vascular involvement in patients with pancreatic cancer is 0.6880 (P = 0.0531). The sensitivity is 70.37%, and the specificity is 60.87% with a cutoff value of 3.9. (B) The area under the ROC curve for evaluating lymph node metastasis is 0.9064 (P < 0.001). The sensitivity is 80%, and the specificity is 81.48% when the critical value is 3.57.

For vascular involvement, serum DRAIC demonstrated a moderate discriminatory capacity, with an AUC of 0.688 (P = 0.0531). Although sensitivity and specificity were 70.4% and 60.9%, respectively, the borderline statistical significance indicates that the predictive value for vascular invasion should be interpreted cautiously (Figure 3A). In contrast, serum DRAIC demonstrated stronger predictive ability for lymph node metastasis, with an AUC of 0.906 (P<0.001), sensitivity of 80%, and specificity of 81.5% at a cutoff value of 3.57 (Figure 3B).

### Postoperative changes in serum DRAIC levels

**Figure 4:**
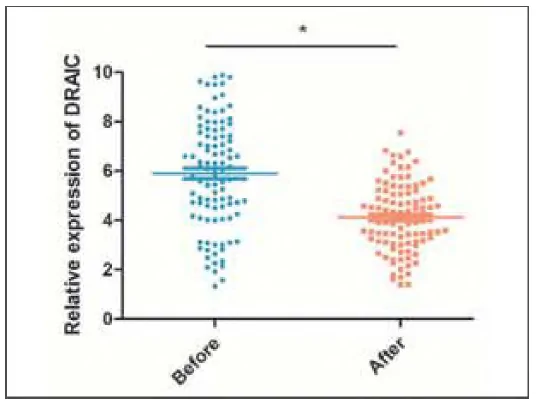
Changes in DRAIC levels after surgery. RT-PCR showed that the expression of DRAIC in postoperative patients was significantly lower than that in preoperative patients.

Serum DRAIC expression levels were measured in all 100 patients before and after surgical treatment. Quantitative PCR analysis showed that serum DRAIC levels after surgery were significantly lower than preoperative levels (Figure 4), indicating a marked reduction following tumour resection.

## Discussion

PC remains among the most aggressive malignancies and is characterised by rapid progression, early dissemination, and extremely poor prognosis [19][20]. Because sensitive and specific biomarkers for early detection are lacking, most patients are diagnosed at advanced stages and often lose the opportunity for curative surgical intervention. Moreover, the therapeutic efficacy of chemotherapy and radiotherapy remains limited, resulting in only modest improvements in long-term survival over recent decades. Consequently, identifying novel molecular and biochemical markers that reflect tumour presence and metastatic potential is critical for improving the clinical management of PC [21].

With the advancement of genomics and transcriptomics, non-coding RNAs participate in diverse biological processes [22][23]. Importantly, non-coding RNAs can modulate gene expression either directly or through complex interaction networks involving other RNA species and proteins, thereby influencing tumour initiation, progression, and metastasis [24][25].

LncRNAs were initially regarded as transcriptional by-products without biological significance [26]. However, subsequent studies have demonstrated that many lncRNAs exhibit tissue-specificity and spatiotemporal expression patterns, and exert functional roles in regulating cellular proliferation, apoptosis, invasion, and metabolic homeostasis [27]. High-throughput biochip technologies and bioinformatics analyses have become essential tools for identifying dysregulated lncRNAs and predicting their biological functions in cancer [28]. For instance, Yang et al. [28] demonstrated that lncRNA HEIH enhances tumour growth in hepatocellular carcinoma by recruiting PRC2 and modulating cell-cycle-related gene expression, highlighting the close link between lncRNA dysregulation and oncogenic signalling pathways.

In pancreatic cancer, several lncRNAs have been implicated in tumour progression and metastasis. Differential expression between primary and metastatic PC tissues has been reported [16], while elevated HOTAIR correlates with poor prognosis and GAS5 acts as a tumour suppressor [29][30]. These findings collectively suggest that lncRNAs have regulatory roles in PC biology and may serve as clinically relevant molecular indicators.

DRAIC, a 1.7 kb lncRNA initially identified in prostate cancer, is aberrantly expressed across multiple malignancies and associated with tumour stage, metastatic behaviour, and treatment response [17][31][32]. In this study, serum DRAIC levels were significantly higher in PC patients than in healthy controls. From a biochemical perspective, this dysregulation suggests that DRAIC may reflect tumour-associated transcriptional reprogramming and systemic molecular alterations associated with PC development. The high area under the ROC curve indicates that circulating DRAIC demonstrates strong diagnostic discrimination, supporting its potential as a serum-based biomarker.

Notably, elevated serum DRAIC levels significantly correlated with lymph node metastasis and vascular involvement. ROC analysis revealed that DRAIC showed strong predictive performance for lymph node metastasis, whereas its predictive value for vascular invasion was moderate. These results indicate that DRAIC may be more closely linked to mechanisms underlying lymphatic dissemination rather than direct vascular infiltration. From a biochemical standpoint, this distinction may reflect differential regulatory roles of lncRNAs in cell-cell adhesion and microenvironmental interactions, although the precise molecular pathways require further investigation.

Although elevated serum DRAIC levels were associated with both lymph node metastasis and vascular involvement, ROC analysis revealed marked heterogeneity in predictive performance. DRAIC demonstrated intense discrimination for lymph node metastasis but only moderate and statistically borderline performance for vascular involvement. These findings suggest that circulating DRAIC may be more closely associated with lymphatic dissemination than with direct vascular infiltration. Accordingly, DRAIC should be considered an adjunct biomarker, particularly for lymph node metastasis, rather than a standalone predictor of vascular invasion.

Surgical resection remains a cornerstone of PC treatment, and reliable biochemical markers that reflect tumour burden and treatment response are of considerable clinical interest. The observed postoperative reduction in serum DRAIC levels further supports its association with tumour presence and demonstrates that DRAIC serves as a dynamic biomarker for monitoring therapeutic efficacy. Nevertheless, given its moderate predictive performance for vascular involvement and the absence of mechanistic validation, DRAIC should currently be regarded as an adjunct rather than a standalone biomarker.

Several limitations should be acknowledged. This was a single-centre study with a moderate sample size, and neither internal validation nor multivariable modelling was performed. The use of GAPDH as a serum reference gene, while supported by prior studies, warrants further validation with additional endogenous or exogenous controls. Moreover, comparisons with established biomarkers such as CA19-9 were not available. Future multicenter studies integrating biochemical, clinical, and mechanistic analyses are required to confirm and extend these findings.

## Conclusion

In conclusion, serum lncRNA DRAIC is significantly dysregulated in patients with PC and has potential utility as a circulating biomarker, particularly for evaluating lymph node metastasis. Although its predictive value for vascular invasion is moderate, postoperative reductions in DRAIC levels further support its association with tumour burden. These findings highlight the clinical and biochemical relevance of DRAIC in PC and provide a basis for future mechanistic and validation studies.

## Dodatak

### Conflict of interest statement

All the authors declare that they have no conflict of interest in this work.
